# Epigenetic regulation of Parkinson’s disease risk variant GPNMB cg17274742 methylation by sex and exercise from Taiwan Biobank

**DOI:** 10.3389/fnagi.2023.1235840

**Published:** 2023-09-07

**Authors:** Yen-Chung Chen, Yi-Chia Liaw, Oswald Ndi Nfor, Chih-Hsuan Hsiao, Ji-Han Zhong, Shey-Lin Wu, Yung-Po Liaw

**Affiliations:** ^1^Department of Public Health and Institute of Public Health, Chung Shan Medical University, Taichung, Taiwan; ^2^Department of Neurology, Changhua Christian Hospital, Changhua, Taiwan; ^3^Neurological Institute, Taipei Veterans General Hospital, Taipei, Taiwan; ^4^Department of Electrical Engineering, Changhua National University of Education, Changhua, Taiwan; ^5^Department of Medical Imaging, Chung Shan Medical University Hospital, Taichung, Taiwan; ^6^Institute of Medicine, Chung Shan Medical University, Taichung, Taiwan

**Keywords:** Parkinson’s disease, DNA methylation, epigenesis, genetic, exercise, sex, gender

## Abstract

**Background:**

Parkinson’s disease (PD) is a complex neurodegenerative disease with an elusive etiology that involves the interaction between genetic, behavioral, and environmental factors. Recently, epigenetic modifications, particularly DNA methylation, have been recognized to play an important role in the onset of PD. Glycoprotein non-metastatic melanoma protein B (GPNMB), a type I transmembrane protein crucial for immune cell activation and maturation, has emerged as a potential biomarker for the risk of PD. This research aims to investigate the influence of exercise and gender on the regulation of methylation levels of GPNMB cg17274742 in individuals.

**Methods:**

We analyze data from 2,474 participants in the Taiwan Biobank, collected from 2008 and 2016. Methylation levels at the *GPNMB* cg17274742 CpG site were measured using Illumina Infinium MethylationEPIC beads. After excluding individuals with incomplete data or missing information on possible risk factors, our final analysis included 1,442 participants. We used multiple linear regression models to assess the association between sex and exercise with adjusted levels of *GPNMB* cg17274742 for age, BMI, smoking, drinking, coffee consumption, serum uric acid levels, and hypertension.

**Results:**

Our results demonstrated that exercise significantly influenced the methylation levels of *GPNMB* cg17274742 in males (β = −0.00242; *p* = 0.0026), but not in females (β = −0.00002362; *p* = 0.9785). Furthermore, male participants who exercised showed significantly lower levels of methylation compared to the reference groups of the female and non-exercising reference groups (β = −0.00357; *p* = 0.0079). The effect of the interaction between gender and exercise on the methylation of *GPNMB* cg17274742 was statistically significant (*p* = 0.0078).

**Conclusion:**

This study suggests that gender and exercise can modulate *GPNMB* cg17274742, with hypomethylation observed in exercise men. More research is needed to understand the underlying mechanisms and implications of these epigenetic changes in the context of risk and prevention strategies.

## Introduction

Parkinson’s disease (PD) is a common neurodegenerative disorder characterized by progressive loss of dopaminergic neurons in the substantia nigra and accumulation of α-synuclein within Lewy bodies (Alam and Schmidt, 2002). PD manifests severe motor and non-motor symptoms that significantly affect the quality of life of patients and caregivers. In particular, less than 10% of cases of PD can be attributed to identified genetic causes (Farrer, 2006). The etiology of idiopathic Parkinson’s disease remains largely enigmatic; however, it is postulated that a combination of genetic, behavioral, and environmental factors contribute to its development (Blauwendraat et al., 2020).

Recently, a growing body of evidence has suggested that epigenetic modifications, particularly DNA methylation, may play a crucial role in the development and progression of PD and could play a role in pathogenesis including α-synuclein misfolding and aggregation, mitochondrial dysfunction, impaired protein clearance, neuroinflammation, and oxidative stress (Pieper et al., 2008; Masliah et al., 2013; Coupland et al., 2014; Su et al., 2015; Eryilmaz et al., 2017; Jankovic and Tan, 2020; Kia et al., 2021). DNA methylation, a biochemical modification that occurs predominantly at cytosine residues in CpG dinucleotides, can influence gene expression and contribute to the complex regulation of cellular processes (Gius et al., 2004). As aging progresses, the accumulation of errors in the epigenetic machinery increases the risk of age-related pathologies, such as brain deterioration and neurodegeneration (Salameh et al., 2020). Aberrant DNA methylation has been implicated in the dysregulation of various molecular pathways associated with PD, highlighting the importance of further investigating the role of epigenetics in the context of the pathogenesis of PD (Renani et al., 2019; Kia et al., 2021).

Glycoprotein non-metastatic melanoma protein B (GPNMB) is a type I transmembrane protein that plays a key role in immune cell maturation and activation. It has been closely associated with PD as a potential risk biomarker and in modulating neuroinflammation, a hallmark of the pathogenesis of PD (Jankovic and Tan, 2020; Saade et al., 2021; Diaz-Ortiz et al., 2022; Kaiser et al., 2023). Recent studies have shown an increase in GPNMB expression within the substantia nigra of PD patients, suggesting a risk factor through its interaction with alpha-synuclein (Moloney et al., 2018; Diaz-Ortiz et al., 2022). Recent evidence on the causality and heterogeneity of PD suggests that GPNMB is the main causal protein in the high-throughput proteomic analysis of cerebrospinal fluid (Kaiser et al., 2023). Interestingly, overexpression of GPNMB in animal models has been shown to reduce dopaminergic neuron degeneration and exert an anti-neuroinflammatory effect (Neal et al., 2018; Budge et al., 2020). Genome-wide association studies (GWAS) have also discovered single nucleotide polymorphisms (SNPs) within the *GPNMB* gene, which can increase the risk of PD and could be regulated by DNA methylation. Specifically, the CpG site, located in the exon region of the *GPNMB* gene on chromosome 7, is associated with the methylation status of the *GPNMB* gene (International Parkinson’s Disease Genomics Consortium (IPDGC), and Wellcome Trust Case Control Consortium 2 (WTCCC2), 2011; Nalls et al., 2014; Kia et al., 2021).

The prevalence of Parkinson’s disease varies significantly by sex, with a higher incidence observed in men than in women. This gender difference can be attributed to hormonal, genetic, and lifestyle factors (Picillo et al., 2017; Russillo et al., 2022). Several lifestyle and behavioral choices are associated with gender differences (Bellou et al., 2016; Cerri et al., 2019), and among lifestyle factors, exercise is one of the well-studied factors believed to alter DNA methylation (Daniele et al., 2018; Ferioli et al., 2019; Xu et al., 2021). Exercise can also mitigate the progression of PD, with neuroinflammatory modulation suggested as a key mechanism (Chen et al., 2005; Crotty and Schwarzschild, 2020; Xu et al., 2021; Sujkowski et al., 2022). However, complex epigenetic changes related to exercise and different sexes in PD are not fully understood.

Currently, no studies have examined the influence of sex and exercise on GPNMB expression and DNA methylation. Given that both epigenetic alterations and lifestyle factors can affect GPNMB expression and contribute to the pathogenesis of PD, this research could provide valuable information on the progression of the disease and possible interventions. Using DNA methylation data, precision public health can facilitate personalized prevention for people at risk of developing Parkinson’s disease, such as providing specific physical activity guidelines based on the individual’s unique methylation profile. Therefore, a comprehensive assessment of the impact of gender and exercise on GPNMB expression and DNA methylation status in PD could have significant implications for precision public health strategies in PD.

## Materials and methods

### Data source and participants

Data for this study were sourced from Taiwan Biobank (TWB), an ongoing prospective cohort study that includes more than 150,000 participants. The TWB contains demographic and whole genome sequencing data from Taiwanese (99% Han Chinese) between 20 and 70 years of age who do not have a cancer diagnosis, with lifestyle information obtained through individual interviews (Chen et al., 2016; Wei et al., 2021).

Anthropometric and biochemical data was collected from the medical centers that enroll subjects. Initially, 2,474 participants were enrolled in this study. After the exclusion of patients with incomplete data (*N* = 1,032), the final sample for analysis consisted of 1,442 subjects.

This study was approved by the Institutional Review Board of the Chung Shan Medical University Hospital (CS1-20009).

### DNA methylation

Methylation profiles were analyzed with the Infinium MethylationEPIC BeadChip Kit (Illumina Inc., San Diego, CA, United States) (Shen et al., 2007; Irizarry et al., 2008; du et al., 2010), which targets more than 850,000 CpG sites. Epigenetic data was obtained from whole blood samples. Epigenetic data was extracted from whole blood. We adjust the cell-type composition using the Reference-Free Adjustment for Cell-Type Composition (ReFACTor) method (Chen et al., 2016). Methylation levels were quantified using a beta value (0–1) to present hypo/hypermethylation. Beta value of the CpG site within the exon region of the *GPNMB* gene.

### Outcome definition and covariates

The beta value of GPNMB cg17274742 for each participant. Body mass index (BMI) (kg/m2) was calculated as body weight (kilogram) divided by body height (meter) squared. The BMI classifications were the following: underweight (0 to 18.5), normal (18.5 to 24), overweight (), and obese (more than 27). For this study, people who exercised for more than 30 min at least three times a week were considered to have regular exercise habits. The waist-hip ratio (WHR) is derived by dividing the waist circumference by the hip circumference. Current drinkers were classified as individuals who consumed more than 150 mL/week in the previous 6 months, while former drinkers had abstained for at least 6 months. Current smokers were identified as individuals who smoked continuously within the last 6 months, and former smokers had resisted smoking for at least 6 months. Coffee consumption was classified into ‘no / yes’ groups. A positive history of hypertension was determined in the self-report of a physician’s diagnosis. Taiwan Biobank has commissioned the Linkou Branch of Chang Gung Memorial Hospital to supervise blood and urine analyzes. Within the scope of serological assessments, particular emphasis is placed on the quantification of uric acid, with a reference threshold established at <7 mg/dL.

### Statistical analysis

To investigate the association between methylation in *GPNMB* cg17274742 and sex and exercise status, multiple linear regression was used after adjusting for age, BMI, smoking status, alcohol consumption, coffee intake, serum uric acid level, and hypertension. The interaction between sex and exercise was also considered in the model. Statistical significance was established with a value of *p* <0.05 in a two-tailed test. Differences were evaluated using the t-test for continuous variables and the chi-square test for categorical variables. All analysis procedures were performed using the SAS 9.4 software version (SAS Institute Inc., Cary, NC, United States).

## Results

The analysis included a total of 1,442 subjects (men = 690, women = 752). The average age of the participants was similar between sexes (48.73 ± 0.39 for women, 49.66 ± 0.43 for men, *p*-value = 0.1121) (Table 1). The level of methylation of *GPNMB* cg17274742 (beta value) did not show significant differences between men and women (0.9480 ± 0.000401 in men, 0.9487 ± 0.000428 in women, *p*-value = 0.2257). Men exhibited a higher proportion of regular exercise (47.1% in men, 40.7% in women, *p* = 0.0142), higher BMI (61.6% in men, 37.3% in women, *p* < 0.0001), higher prevalence of smoking (42.7% in men, 6.4% in women, *p* < 0.0001), alcohol consumption (17.8% in men, 2.3% in women, *p* < 0.0001), history of hypertension (17% in men, 8.1% in women, *p* < 0.0001), and higher serum uric acid levels (6.34 mg/dL in men, 4.76 mg/dL in women, *p* < 0.0001). Coffee consumption was comparable between sexes. The WHR for women is manifested as 0.8411 ± 0.003, while for men, it registers at 0.8931 ± 0.002.

**Table 1:** Demographic characteristics.

	Female (*n* = 752)	Male (*n* = 690)	*p*-value
*methylation of cg17274742 methylation (beta value)*	0.9487 ± 0.000428	0.9480 ± 0.000401	0.2257
*Exercise (n, %)*			0.0142
No	446 (59.31)	365 (52.90)	
Yes	306 (40.69)	325 (47.10)	
*Age (years)*	48.73 ± 0.39	49.66 ± 0.43	0.1121
*Body Mass Index* (*n*, %)			<0.0001
Normal	439 (58.38)	259 (37.54)	
Underweight	32 (4.26)	6 (0.87)	
Overweight	169 (22.47)	251 (36.38)	
Obese	112 (14.89)	174 (25.22)	
*Smoking (n, %)*			<0.0001
Never	704 (93.62)	395 (57.25)	
Former	27 (3.59)	169 (24.49)	
Current	21 (2.79)	126 (18.26)	
*Drinking (n, %)*			<0.0001
Never	735 (97.74)	567 (82.17)	
Former	6 (0.80)	40 (5.80)	
Current	11 (1.46)	83 (12.03)	
*Coffee (n, %)*			0.8008
No	478 (63.56)	443 (64.20)	
Yes	274 (36.44)	247 (35.80)	
*Serum Uric Acid Level (mg/dL)*	4.7641 ± 0.0388	6.3399 ± 0.0503	<0.0001
*Hypertension (n, %)*			<0.0001
No	691 (91.89)	573 (83.04)	
Yes	61 (8.11)	117 (16.96)	

Data are displayed as mean ± standard error (SE) or numbers (percentage).

The regression model revealed that being male was associated with a significantly lower level of *GPNMB* cg17274742 than being female (β = −0.00252; *p*-value = 0.0417) after adjusting for possible confounders (Table 2). Furthermore, patients with hypertension showed a higher beta value, indicative of hypermethylation. Regular exercise was not associated with the level of methylation (β = −0.00108; *p*-value = 0.0681).

**Table 2:** The association of sex and exercise with the methylation of cg17274742.

	β	*p*-value
*Sex (ref: female)*		
Male	−0.00252	0.0417
*Exercise (ref: no)*		
Yes	−0.00108	0.0681
*Age (years)*	0.00005839	0.1413
*Body Mass Index (ref: normal)*		
Underweight	−0.00309	0.0716
Overweight	0.00047922	0.4660
Obese	−0.00051091	0.5017
*Smoking (ref: never)*		
Former	0.00007943	0.9261
Current	−0.00023201	0.8152
*Drinking (ref: never)*		
Former	−0.00209	0.1837
Current	0.00026805	0.8146
Coffee (ref: no)		
Yes	0.00024941	0.6576
*Serum Uric Acid Level (mg/dL)*	0.00004080	0.8617
*Hypertension (ref: no)*		
Yes	0.00183	0.0332

β, beta value.

A significant interaction was observed between sex and exercise on the methylation of *GPNMB* cg17274742 (*p*-value = 0.0078). Stratified analysis by sex (Table 3) revealed that exercise was associated with *GPNMB* cg17274742 only in men (β = −0.00242; *p*-value = 0.0026) and not in women (β = −0.00002362; *p*-value = 0.9785). When stratified by exercise, no significant association was found for either sex group (Table 4). When considering sex and exercise together, men who exercised regularly exhibited significantly lower levels of *GPNMB* cg17274742 compared to women who did not exercise regular exercise (β = −0.00357; *p*-value = 0.0079) (Table 5).

**Table 3:** The association of exercise with methylation of cg17274742 stratified by sex.

	Female		Male	
	β	*p*-value	β	*p*-value
*Exercise (ref: no)*				
Yes	−0.00002362	0.9785	−0.00242	0.0026
*Age (years)*	0.00009677	0.1070	0.00003282	0.5372
*Body Mass Index (ref: normal)*				
Underweight	−0.00358	0.0702	0.00070555	0.8602
Overweight	−0.00005231	0.9580	0.00104	0.2326
Obese	−0.0014	0.2306	0.00040407	0.6896
*Smoking (ref: never)*				
Former	0.00238	0.2590	−0.00027879	0.7595
Current	−0.00002935	0.9903	−0.00018952	0.8592
*Drinking (ref: never)*				
Former	0.00564	0.2009	−0.00254	0.1203
Current	0.00431	0.1863	−0.00036559	0.7584
*Coffee (ref: no)*				
Yes	−0.00036098	0.6603	0.00105	0.1756
*Serum Uric Acid Level (mg/dL)*	−0.00011601	0.7639	0.00001066	0.9713
*Hypertension (ref: no)*				
Yes	0.00282	0.0573	0.00119	0.2530

Interaction (sex * exercise) *p*-value = 0.0078. β, beta value.

**Table 4:** The association of sex with stratified methylation of cg17274742 by exercise.

	No-Exercise		Exercise	
	β	*p*-value	β	*p*-value
*Sex (ref: female)*				
male	−0.00243	0.1580	−0.00248	0.1717
*Age (years)*	0.00004747	0.3758	0.00006807	0.2581
*Body Mass Index (ref: normal)*				
underweight	−0.00542	0.0073	0.00341	0.3024
overweight	0.00059352	0.5139	0.00037876	0.6923
obese	−0.00101	0.3050	0.00037229	0.7615
*Smoking (ref: never)*				
former	0.00021207	0.8571	−0.00011083	0.9304
current	−0.00046354	0.7037	−0.00016023	0.9269
*Drinking (ref: never)*				
former	−0.00154	0.4813	−0.00153	0.5054
current	0.00003673	0.9808	0.00043187	0.8045
*Coffee (ref: no)*				
yes	0.00015507	0.8369	0.00063038	0.4647
*Serum Uric Acid Level (mg/dL)*	0.00008704	0.7812	0.00001214	0.9729
*Hypertension (ref: no)*				
yes	0.00155	0.2120	0.00207	0.0847

Interaction (sex * exercise) *p*-value = 0.0078. β, beta value.

**Table 5:** The association of cg17274742 methylation is based on sex and exercise.

	β	*p*-value
*Sex and exercise (ref: female and no exercise)*		
Female and exercise	0.0003421	0.6663
Male and no-exercise	−0.00099486	0.4645
Male and exercise	−0.00357	0.0079
*Age (years)*	0.00006072	0.1254
*Body Mass Index (ref: normal)*		
Underweight	−0.00298	0.0811
Overweight	0.00043034	0.5120
Obese	−0.0005592	0.4614
*Smoking (ref: never)*		
Former	0.00017939	0.8339
Current	−0.00031185	0.7530
*Drinking (ref: never)*		
Former	−0.00195	0.2145
Current	0.00024349	0.8310
*Coffee (ref: no)*		
Yes	0.00034958	0.5345
*Serum Uric Acid Level (mg/dL)*	0.00002553	0.9130
*Hypertension (ref: no)*		
YES	0.00186	0.0296

β, beta value.

## Discussion

Using data from a large prospective cohort, we discerned a sex-specific association between exercise and methylation status. Our findings suggest that men who exercise regularly have a lower level of methylation of cg17274742, which can result in increased expression of GPNMB. Furthermore, we can discern a tangible impact of exercise on methylation patterns at the cg17274742 locus, particularly within the male cohort. Men who exercise are observed to experience a significant reduction in methylation at this genomic site. When these data are juxtaposed against the corresponding female cohort, a stark contrast emerges. Within the exercise-engaged population, males show a more pronounced decrease in methylation relative to females. These findings not only reveal a complex interplay between physical exercise and DNA methylation, but also underscore the need to consider gender-specific variations in the field of epigenetic research. This is the first study to find a relationship between different sexes and exercise-associated methylation changes, suggesting that a lifestyle change, particularly a routine exercise habit in men, could increase the expression of GPNMB, which is highly correlated with Parkinson’s disease and the anti-inflammatory reaction.

GPNMB is a protein that is found in cell membranes of various tissues, including the nervous system, skin, and bone (Lazaratos et al., 2022). Previous studies have noted the importance of different biological processes and functions of GPNMB, including cell differentiation and development, inflammation and immune response, progression, and neurodegeneration deterioration (Zhang et al., 2017; Neal et al., 2018; Budge et al., 2020; Saade et al., 2021; Diaz-Ortiz et al., 2022; Lazaratos et al., 2022). GPNMB can have neuroprotective effects in mitigating the effects of harmful protein aggregates, a common feature in many neurodegenerative diseases (Tanaka et al., 2012; Budge et al., 2018; Zhu et al., 2022). Gpnmb levels are a reliable indicator of the severity of the disease in various medical conditions. When Gpnmb is up-regulated, it is commonly associated with infiltration of antigen-presenting cells into the affected tissue (Maric et al., 2013; Suda et al., 2022). Initially, significant up-regulation is often interpreted as disease-related and harmful. However, it is essential to consider that up-regulation of Gpnmb may result from macrophages that exert anti-inflammatory and immune-balancing effects, possibly through interactions with T cells. Cancer cells may take advantage of this immune-dampening property of Gpnmb to enhance their progression. The role of Gpnmb in different diseases, especially neurodegenerative diseases, becomes complex, particularly due to potential variations in its function when bound to cells or present in a soluble form (Tanaka et al., 2012; Neal et al., 2018; Xie et al., 2019; Saade et al., 2021; Zhu et al., 2022).

Recent studies highlight the importance and benefits of involving diverse and multi-ethnic populations in genetic studies (Popejoy and Fullerton, 2016; Graham et al., 2021) including a more accurate representation of the risks of genetically associated diseases in different populations (Sirugo et al., 2019). To our knowledge, most published genome-wide association studies (GWAS) and methylomic investigations involving GPNMB variants were conducted predominantly with Caucasian populations (Nalls et al., 2014; Chang et al., 2017; Murthy et al., 2017; Iwaki et al., 2019; Prasad and Jho, 2019; Langmyhr et al., 2021). Our study used information from the Taiwan Biobank (TWB), the largest biobank in East Asia, known for its high-coverage whole genome sequencing and DNA methylation data in a Han Chinese population (Wei et al., 2021).

A recent comprehensive strategy aims to colocalize the expression quantitative trait loci (eQTL) and GWAS signals in PD using an updated PD GWAS dataset. The methylation study identified cg17274742 in the GPNMB gene, which has implications for Coloc expression, which evaluates shared causal variants between eQTL and GWAS signals. Methylation has an impact on gene expression, and specifically cg17274742 in GPNMB influences not only expression but also the splicing function at the *GPNMB* (gene level)/ *NUPL2* (splicing) locus through strong protein–protein interactions evidence that connects to mendelian or sporadic risk genes (Kia et al., 2021). PD-associated variants exert their influence on GPNMB through this methylation site, uncovering molecular insights into the mechanisms of PD and underscoring the importance of genetic and epigenetic factors.

We observed a predominant hypomethylation of cg17274742 in men, which could lead to an increase in GPNMB expression. After adjusting for age in the epidemiological study, the prevalence of PD in men is approximately 1.4 times that of women (Dorsey et al., 2018) although a 2014 meta-analysis suggests that this difference is evident only in the age group (Pringsheim et al., 2014). The sex differences in *GPNMB* cg17274742 observed in our study could partially explain this prevalence disparity in PD. Hormonal variances, especially estrogen levels, can contribute to risk and progression, given the neuroprotective effects demonstrated in animal models (Russillo et al., 2022). The intricate interplay of sex hormones, such as estrogens, manifests itself as a modulatory influence on humoral immunity, effectively improving its response. In contrast, androgens, progesterone, and glucocorticoids exhibit inherent immunosuppressive properties that contribute to the regulation and fine-tuning of the immune system. Of particular importance is the heterogeneity of sex steroids, which encompass estrogens, progesterone, and testosterone, which exhibit notable differences between genders and undergo dynamic changes in various reproductive phases. These complex hormonal variations exert a substantial influence on the intricate details of neural function, prompting a critical exploration of their roles in neurological pathophysiology (Bouman et al., 2005). GPNMB protein was also found to be regulated by sex hormones or modulated in a specific sex manner in deep immunophenotyping animal model studies (Tsui et al., 2012; Houser et al., 2022). Recent research has also identified specific sex changes in DNA methylation in brain tissue (Kochmanski et al., 2022).

Furthermore, our findings indicate that hypertension is associated with hypermethylation of cg17274742, which could reduce the expression of GPNMB and therefore possibly lower the risk of PD (Murthy et al., 2017; Diaz-Ortiz et al., 2022). However, the existing literature presents contradictory evidence linking hypertension and the diagnosis of PD, and different effects of hypertension have been reported in Caucasian versus Asian populations (McCann et al., 1998; Paganini-Hill, 2001; Qiu et al., 2011; Hou et al., 2018; Chen et al., 2019; Ng et al., 2021). Additionally, certain antihypertensive medications can influence the risk of PD (Lee et al., 2014; Cai et al., 2019; Simmering et al., 2021; Jo et al., 2022; Lin et al., 2022; Simmering et al., 2022).

Regular exercise is widely recognized to exert a protective influence against PD through mechanisms such as neuroplasticity, angiogenesis, and modulation of oxidative damage and neuroinflammation (Ascherio and Schwarzschild, 2016; Jang et al., 2017; Palasz et al., 2019; Mahalakshmi et al., 2020; Ruiz-González et al., 2021; Mazo et al., 2022). Increasing evidence suggests that exercise can alter brain function and structure, slowing the progression of motor and non-motor symptoms of PD (Reynolds et al., 2016; Schenkman et al., 2018; van der Kolk et al., 2019; Johansson et al., 2022; Tsukita et al., 2022). Exercise can also produce gender-specific effects on gene regulation and expression through epigenetic modifications and hormonal changes (Landen et al., 2019; Wu et al., 2020). Both the innate and adaptive immune systems involve essential cellular components that possess receptors for sex hormones, enabling them to respond to hormonal signals. This intriguing observation suggests that the impact of exercise on the immune system could differ between men and women, highlighting the potential role of sex-dependent factors in mediating immune responses during physical activity (Kadel and Kovats, 2018; Fuentes and Silveyra, 2019; Becerra-Diaz et al., 2020). In a similar vein, the female reproductive cycle can influence changes in the expression of inflammatory genes induced by exercise in various circumstances (Northoff et al., 2008). In addition, there is an ongoing debate about the contrasting effects of different types of exercise on immune responses and related outcomes between men and women (Soligard et al., 2017; Barrett et al., 2018; Drew et al., 2018; Zhou et al., 2018). Furthermore, a large-scale epigenome-wide association meta-analysis suggested that genes with differences in methylation between sexes are also present in human skeletal muscle (Landen et al., 2021).

The first limitations of our research include the focus on carriers of the *GPNMB* variant, rather than individuals with a clinically confirmed diagnosis of Parkinson’s disease. However, the potential influence of methylation alterations on gene expression, compounded by environmental and lifestyle variables, underscores the importance of precision public health in our investigative approach. To capitalize on this concept, our study aims to elucidate the lifestyle determinants that affect gene carriers during the incipient stages of the disease. Through these insights, we anticipate the development of suitable interventions that can postpone the onset of the disease. The second source of uncertainty is that according to the Taiwan Human Biobank, inquiries related to alcohol consumption only address drinking habits and do not include questions pertaining to the quantification of alcohol intake, and only address the frequency of smoking, without encompassing questions related to the total volume of cigarette consumption. The other constraint of our study lies in the inability to fully account for all possible confounders, such as diet, lifestyle, and environmental exposure. However, we adjusted for factors such as age, BMI, smoking, drinking, coffee consumption, serum uric acid levels, and hypertension, which are widely recognized as potential risk or protective factors based on extensive epidemiological studies. Although our research offers valuable information on the relationship between exercise and DNA methylation between sexes, the findings should be interpreted with caution due to the limitations of the study. Future experimental studies could potentially confirm our findings using animal models, further elucidating the causal relationship between exercise and DNA methylation of the *GPNMB* gene in different sexes.

Ultimately, our results provide epigenetic insight into the sex and lifestyle factors associated with GPNMB expression, which served as a potential biomarker and therapeutic target for PD. DNA methylation presents a promising avenue for the application of precision public health in the treatment of PD, including personalized diet recommendations and exercise programs for different sexes based on an individual methylation profile. More research is needed to fully understand the intricate relationships between lifestyle factors, DNA methylation, and the progression of PD.

## Data availability statement

The data analyzed in this study is subject to the following licenses/restrictions: Data supporting the findings of this study are available from the Taiwan Biobank, but restrictions apply to the availability of these data, which were used under license for the current study and are not publicly available. However, the data are available from the authors upon reasonable request and with permission of Taiwan Biobank. Requests to access these datasets should be directed to Y-PL, liawyp@csmu.edu.tw.

## Ethics statement

The studies involving humans were approved by the Institutional Review Board of the Chung Shan Medical University Hospital (CS1-20009). The studies were conducted in accordance with the local legislation and institutional requirements. The participants provided their written informed consent to participate in this study.

## Author contributions

Y-CC, S-LW, and Y-PL: conceptualization. C-HH and J-HZ: data curation and formal analysis. ON, Y-CC, and Y-PL: methodology. S-LW and Y-PL: supervision. Y-CC: writing – original draft. Y-CC and Y-CL: writing-review & editing. All authors contributed to the article and approved the submitted version.

## Funding

This work was funded by the Ministry of Science and Technology (MOST 109-2121-M-040-002; 109-2811-M-040-500; 110-2811-M-040-001; 110- 2121-M-040-002; 111-2811-M-040-001; 111-2121-M-040-002; 111-2811-M- 040-001).

## Conflict of interest

The authors declare that the research was conducted in the absence of any commercial or financial relationships that could be construed as a potential conflict of interest.

## Publisher’s note

All claims expressed in this article are solely those of the authors and do not necessarily represent those of their affiliated organizations, or those of the publisher, the editors and the reviewers. Any product that may be evaluated in this article, or claim that may be made by its manufacturer, is not guaranteed or endorsed by the publisher.
